# Anti-tumor activity of resveratrol against gastric cancer: a review of recent advances with an emphasis on molecular pathways

**DOI:** 10.1186/s12935-021-01773-7

**Published:** 2021-01-21

**Authors:** Milad Ashrafizadeh, Hossein Rafiei, Reza Mohammadinejad, Tahereh Farkhondeh, Saeed Samarghandian

**Affiliations:** 1 Faculty of Engineering and Natural Sciences, Sabanci University, Orta Mahalle, Üniversite Caddesi No. 27, Orhanlı, Tuzla, Istanbul 34956 Turkey; 2 Sabanci University Nanotechnology Research and Application Center (SUNUM), Tuzla, Istanbul 34956 Turkey; 3 Department of Biology, Faculty of Sciences, Shiraz Branch, Islamic Azad University, Shiraz, Iran; 4 Pharmaceutics Research Center, Institute of Neuropharmacology, Kerman University of Medical Sciences, Kerman, Iran; 5 Medical Toxicology and Drug Abuse Research Center (MTDRC), Birjand University of Medical Sciences, Birjand, Iran; 6 Faculty of Pharmacy, Birjand University of Medical Sciences, Birjand, Iran; 7 Noncommunicable Diseases Research Center, Neyshabur University of Medical Sciences, Neyshabur, 9318614139 Iran

**Keywords:** Resveratrol, Gastric cancer, Cell signaling, Cancer prevention, Anti‐tumor activity, Molecular pathways

## Abstract

Gastric cancer (GC) is one of the most common cancers with high malignancy. In spite of the great development in diagnostic tools and application of anti-tumor drugs, we have not witnessed a significant increase in the survival time of patients with GC. Multiple studies have revealed that Wnt, Nrf2, MAPK, and PI3K/Akt signaling pathways are involved in GC invasion. Besides, long non-coding RNAs and microRNAs function as upstream mediators in GC malignancy. GC cells have acquired resistance to currently applied anti-tumor drugs. Besides, combination therapy is associated with higher anti-tumor activity. Resveratrol (Res) is a non-flavonoid polyphenol with high anti-tumor activity used in treatment of various cancers. A number of studies have demonstrated the potential of Res in regulation of molecular pathways involved in cancer malignancy. At the present review, we show that Res targets a variety of signaling pathways to induce apoptotic cell death and simultaneously, to inhibit the migration and metastasis of GC cells.

## Introduction

Cancer is considered as the most challenging public health issue in both developing and developed countries [1–4]. This life threatening condition burdens high socioeconomic cost. It seems that the incidence rate of cancer is rapidly growing due to the aging of population [5, 6]. Over the past decades, we have witnessed an increase in the incidence rate of Gastric cancer (GC), so that estimates demonstrate that up to 1 million new cases of GC are diagnosed annually and over 700.000 deaths occur [7–11]. This has resulted in much attention towards this cancer. Epidemiological studies demonstrate that GC occurs with high frequency in Asia, Europe, and South America [12]. The World Health Organization (WHO) has divided GC into four characteristic categories including papillary, mucinous, tubular, and signet ring cell [13]. To date, several diagnostic tools have been developed for GC. The most challenging barrier in GC therapy is the diagnosis of this life-threatening condition at advanced stages. Diagnostic tools have enabled us to diagnose GC in its early stages and subsequently, its elimination. Endoscopic ultrasound, computed tomography (CT), magnetic resonance imaging [14], and positron emission tomography are the most common diagnostic tools used in GC diagnosis [15].

Hereditary factors are responsible for about 1–3 % of cancer, while environmental factors are the main reasons of cancer. Smoking, lack of exercise, and poor diet are the major environmental factors of cancer [12, 16]. Much attention has been directed towards cancer therapy and using chemotherapeutic agents is of interest. However, in spite of application of a high amount of chemotherapeutic agents, we have not witnessed a remarkable increase in the survival time of patients with cancer. This has led to the looking at nature as a rich source of anti-tumor drugs. Several studies have revealed the great potential of plant-derived chemicals in inhibition of proliferation and migration of cancer cells, stimulation of apoptotic and autophagic cell death, and enhancing the efficacy of chemotherapy [17–20]. Resveratrol (Res) as a naturally occurring compound, is considered a secondary metabolite exclusively derived from plants and microbial sources [21–23]. The synthesis process of Res is triggered by the action of stilbene synthase (STS) enzyme that incorporates three malonyl coenzyme-A units into 4-hydroxycinnamoyl-CoA (p-coumaroyl-CoA) [24]. This non-flavonoid polyphenol compound is present in a number of plants including grapes, peanuts, and berries [25–27]. A growing body of evidence demonstrates that Res functions as a part of defense system of plants responding to insect and pathogen attacks [28, 29]. Besides, Res is capable of protecting plants against fungal infections and ultra-violet (UV) radiations [30–33]. Overall, Res is available in two forms known as cis and trans due to the central ethylene moiety. It seems that the major form of Res is trans-isomer. However, exposing to the UV is associated with formation of cis-isomer [34–36]. Accumulating data demonstrates that Res has a variety of pharmacological and health-promoting impacts such as antioxidant [37], anti-inflammatory [38], anti-diabetic [39], anti-tumor [40], hepatoprotective [41], and cardioprotective [42].

The great biological and therapeutic activities of Res have led to its application in treatment of various cancers. It is held that Res is able to target different molecular signaling pathways in cancer therapy. One of the difficulties in cancer therapy is the resistance of tumor cells into chemotherapy. This problem has led to the development of novel synthetic anti-tumor drugs. However, application of the high amount of an anti-tumor drug reduces its capability in next treatments. Furthermore, a number of signaling pathways are involved in dynamic progression of tumor cells demanding combination therapy in suppressing cancer cells. It seems that urokinase-type plasminogen activator receptor (uPAR) contributes to the regulation of epidermal growth factor receptor (EGFR) [43]. Overexpression of uPAR is associated with resistance of cancer cells to chemotherapy. Administration of Res sensitizes oral squamous cell carcinoma (OSCC) to chemotherapy by down-regulation of uPAR and its downstream mediator ERK1/2 [44]. Res is able to regulate microRNAs (miRs) in enhancing the efficacy of chemotherapy. Accumulating data demonstrates that Res upregulates oncosuppressor miR to stimulate apoptotic cell death in cancer cells [45, 46]. Exposure to Res improves the chemotherapy potential by enhancing the expression of miR-122-5p leading to the induction of apoptosis and reduced viability of cancer cells [47]. Epithelial-to-mesenchymal transition (EMT) contributes to the increased malignancy and invasion of tumor cells [48]. Inhibition of EMT is of importance in cancer therapy. Administration of Res remarkably diminishes the proliferation and invasion capabilities of breast and lung cancer cells by stimulation of tumor suppressor Rad9 [49]. These studies highlight this fact that Res is capable of regulation of signaling pathways involved in cancer malignancy [50, 51] and its administration can be considered as a promising strategy in tumor therapy. Notably, various molecular signaling pathways are involved in the malignancy of GC cells and there have been efforts to identify these pathways and also their upstream and downstream mediators. Accumulating data demonstrates that abnormal expression of miRs is associated with development of cancer [52–54]. In the case of GC, a similar story occurs. It seems that GC cells down-regulate the expression of miR-27b-3p to ensure their viability and proliferation through enhancing the expression of GSPT1 [55]. It is held that the PI3K/Akt signaling pathway contributes to the progression of GC cells by EMT stimulation [56, 57]. Importantly, UFM1 is associated with decreased migration of GC cells through inhibition of PI3K/Akt molecular signaling [58]. Long non-coding RNAs (lncRNAs) are non-protein coding RNA molecules with the length of 200 nucleotides. It has been reported that lncRNA deregulation leads to cancer generation [59]. LncRNAs are able to dually reduce/enhance the malignancy of cancer cells. A study reveals that lncRNA HOTAIR is capable of elevating the invasion of GC cells by induction of CXCR4 and RhoA signaling pathways, while another study demonstrates that lncRNA GAS5 is related to the inhibited metastasis of GC cells by targeting p53 [60, 61]. Mitogen-activated protein kinase (MAPK), Wnt, and nuclear factor erythroid 2-related factor 2 (Nrf2) are other molecular pathways involved in GC malignancy [62–64].

At the present review, we demonstrate how Res can be beneficial in treatment of gastric cancer (GC).

## Current therapeutic strategies, challenges and future prospective for gastric cancer

Currently, surgery and chemotherapy are the most common strategies in treatment of GC [65]. However, the recurrence of GC usually occurs after surgery. Besides, the resistance of GC cells into chemotherapy is another major problem. Notably, there have been efforts to improve the efficacy of chemotherapy. A look at newly published articles demonstrates that naturally occurring compounds are applied to enhance the potential of chemotherapy. Curcumin is one of them with high anti-tumor capability [66–68]. Curcumin is able to improve the anti-tumor activity of 5-fluorouracil (5-FU) against GC cells by inhibition of COX-2 and NF-κB signaling pathways [69]. Berberine is another potential anti-tumor agent [70]. [70]. It seems that administration of berberine is associated with enhanced inhibitory impact of EGFR inhibitors on GC cells [71]. It appears that plant-derived chemicals are extensively used in GC therapy. However, there are some challenges faced in GC therapy. The most important one is the low bioavailability of applied anti-tumor drugs. Furthermore, lack of specific targeting leads to the toxicity of anti-tumor drugs against normal cells. Nanoparticles (NPs) are focused to increase the bioavailability of anit-cancer agents such as Res. NPs are structures with a particle size as low as 100 nm. These nanocarriers are able to remarkably enhance the bioavailability of anti-tumor drugs by protection against degradation and also prevention of drug trapping via phagocytosis system. On the other hand, identification of cell membrane receptors on cancer cells have resulted in the development of receptor-targeted nanocarriers and consequently, high anti-tumor activity [55, 72, 73].

### Resveratrol and gastric cancer

#### Resveratrol effect on tumor cell cycle

Although EMT is suggested to be beneficial in wound healing and other physiological processes, this mechanism remarkably enhances the metastasis of tumor cells [83]. During EMT, an increase occurs in the migratory capability of cells via transformation of epithelial cells into mesenchymal cells [84, 85]. Various signaling pathways are involved in EMT and accumulating data demonstrates that metastasis-associated lung adenocarcinoma transcript 1 (MALAT1) is capable of induction of EMT in a number of cancers leading to their high invasion capability [86–88]. It appears that administration of Res effectively down-regulates MALAT1 to prevent EMT resulting in reduced invasion and metastasis of GC cells [64].

Accumulating data demonstrates that the hedgehog (Hh) signaling pathway is vital for physiological conditions such as hematopoiesis and is also involved in tumorigenesis [89, 90]. It has been reported that aberration in Hh signaling pathway occurs in a number of cancers such as lung cancer, prostate cancer and so on [91–95]. Notably, the Hh pathway stimulates EMT in GC [96]. Hence, modulation of this signaling pathway is of importance in inhibition of migration and metastasis of cancer cells. It appears that Gli-1 is a biomarker of abnormal expression of Hh pathway [97]. Administration of Res significantly deactivates Hh pathway by down-regulation of Gli-1. As a result, the expressions of factors involved in EMT such as Snail and N-cadherin undergo down-regulation, while an increase occurs in the expression of E-cadherin to suppress EMT resulting in reduced invasion and migration of GC cells [98].

Accumulating data reveals that Res is able to affect various signaling pathway in treatment of disorders [99, 100]. Down-regulation of protein kinase C (PKC) by Res is related to the reduced viability and growth of cells. It seems that PKC α has high sensitivity to Res administration [101]. It has been demonstrated that PKC σ exerts anti-proliferative and pro-apoptotic impacts [102–104]. Res follows a same strategy in treatment of GC. Administration of Res enhances the expression of cytosolic PKC α and reduces membrane-associated PKC σ protein. These impacts lead to the induction of tumor suppressor p21 and p53. Besides, Res treatment elevates the levels of Fas and Fas-L protein. These effects altogether result in stimulation of cell cycle arrest at G_2_/M phase and trigger apoptotic cell death to suppress GC malignancy [105]. Chemotherapeutic activity of Res mainly depends on its impact on PKC. A growing body of evidence demonstrates that PKC participates in tumor progression, tumor proliferation, tumor viability and tumor migration [106–108]. Res exerts a negligible impact on cell lysis, while it considerably induces G_0_/G_1_ cell cycle arrest and apoptosis by down-regulation of PKC [109] demonstrating the potential role of this signaling pathway in progression and malignancy of GC cells.

Importantly, Res has shown great potential in suppressing the proliferation of tumor cells through targeting cell cycle [110–112]. Res applies various signaling pathways to target cell cycle. It has been demonstrated that Res is capable of affecting the expression of sirtuin 1 (Sirt1) [113, 114]. A same story occurs in GC therapy. Administration of Res stimulates the activation of Sirt1 leading to the cell cycle arrest and induction of senescence in tumor cell of nude mice [115].

#### Resveratrol effect on apoptosis

The stimulation of apoptotic cell death is still one of the most common strategies in the field of cancer therapy. Notably, various molecular signaling pathways contribute to the regulation of apoptosis in cancer cells and identification of these pathways is of importance in cancer therapy [38]. Nuclear factor-κB (NF-κB) is responsible for regulation of immunological responses [116]. A variety of studies have shed some light on the involvement of NF-κB signaling pathway in cancer progression and it seems that NF-κB overexpression is related to the generation of cancer [117–120]. Administration of Res sensitizes cancer cells to apoptosis via NF-κB down-regulation leading to a decrease in the level of anti-apoptotic factor Bcl-2 and an increase in apoptotic factors caspase-3 and caspase-8 [121]. Mitochondria play a significant role in apoptosis induction. As a central gateway, mitochondrial pathway modulates both anti- and pro-apoptotic factors [122–125]. Compounds targeting mitochondria are of interest in cancer therapy by induction of apoptotic cell death [126, 127]. Res uses same strategy in combating GC. Administration of Res is associated with disruption of mitochondrial membrane potential. This leads to the induction of apoptotic cell death through upregulation of caspase-3 and caspase-9, and down-regulation of Bcl-2. Finally, a remarkable decrease occurs in the viability and proliferation of GC cells [128]. Exposing GC cells into Res increases the cells having morphological features of apoptosis such as chromatin condensation, chromatin crescent formation and nucleus fragmentation. Upregulation of BAX and down-regulation of Bcl-2 by Res are involved in these anti-tumor impacts in implanted human primary gastric carcinoma cells in nude mice [129]. Resveratrol plus curcumin could regulate p53 post-translational alterations in rat model of gastric cancer [130].

It is held that various GC cell lines respond differently to the Res administration. A study conducted by Riles and colleagues obviously clarifies this statement. They applied three distinct types of GC cells including AGS, SNU-1 and KATO-III cells. In SUN-1 cells treated with Res, there was no trace of alteration in the expression of mitochondrial-mediated apoptotic proteins such as Bcl-2, BAX, Bid and Smad/Diablo. It seems that survivin inhibition by Res contributes to the reduced viability and proliferation of SUN-1 cells. However, the story is a little different for AGS and KATO-III cells. It appears that mitochondrial dysfunction induced by Res is involved in the stimulation of apoptotic cell death in these cells since an increase occurs in the level of cytochrome C [131]. Regardless of the apoptotic pathway, Res administration is a promising strategy in reducing the migration and malignancy of GC cells [132].

#### Resveratrol effect on inflammation

A growing body of evidence demonstrates that pro-inflammatory cytokines such as interleukin-6 (IL-6) are present with high levels in cancer cells. It seems that enhanced concentration of IL-6 significantly promotes the viability and proliferation of tumor cells [133, 134]. Investigation of molecular signaling pathways shows that IL-6 elevates the progression of cancer cells through induction of Raf-MAPK signaling pathway [135, 136]. Similarly, administration of Res suppresses IL-6-mediated GC invasion through inhibition of Raf-MAPK signaling pathway [137]. The cytokines and peptide growth factors force cells to produce ROS [138, 139]. The ROS generation is a vital step in enhancing the proliferation of cells by acting as intracellular messenger and interacting with molecular pathways such as Ras pathway [140–142]. In respect to the carcinogenesis impact of ROS, using naturally occurring antioxidants such as Res is of interest in cancer therapy. After Res supplementation, an increase occurs in nitric oxide (NO) production by nitric oxide synthase (NOS) induction that interacts with ROS leading to the reduced viability, proliferation and migration of GC cells [143].

#### Resveratrol effect on oxidative stress

As it was mentioned, ROS are considered as potential targets in cancer therapy. It has been demonstrated that enhanced concentration of ROS is associated with a number of pathological conditions [144, 145]. This is due to the adverse impact of Res on the cell membrane and more importantly, genetic material that sensitizes cells to high proliferation and generation of cancer [146]. Although much emphasis was put on the negative role of ROS, it seems that ROS are important elements of homeostasis since they function as second messengers of molecular signaling pathways [147]. Hence, regulation of ROS synthesis is of importance in treatment of pathological conditions and preserving homeostasis. In the case of GC therapy, Res remarkably reduces the concentrations of ROS via its great antioxidant activity. Investigation of molecular pathways demonstrates that inhibition of ROS-mediated GC progression is induced by down-regulation of c-Jun and ERK1/2 phosphorylation through MEK1/2 [148].

#### Resveratrol effect on autophagy

Over the past decades, we have witnessed an attention into autophagy mechanism due to its dual role between life and death [149]. This has resulted in targeting autophagy in cancer therapy [150]. This lysosome-mediated mechanism ensures homeostasis and survival during physiological condition by degradation of aged and damaged organelles and components [70]. Notably, autophagy is involved in caspase-independent programmed cell death [151]. So, autophagic cell death is considered as one of the most promising strategies in cancer therapy [152]. There are a number of pathways and macromolecules that are able to regulate autophagy [73, 153, 154]. Dihydroceramide is a ceramide metabolic precursor involved in sphingolipid synthesis. Dihydroceramide desaturases (Des1 and Des2) convert the dihydroceramide into ceramide. Accumulating data demonstrates that dihydroceramide is capable of induction of autophagy [155, 156]. Res administration significantly enhances the intracellular level of dihydroceramide to trigger autophagy leading to the reduced viability and proliferation of GC cells and sensitizing these malignant cells into apoptosis [157].

Table 1 indicates the potential therapeutic effects of resveratrol against gastric cancer.

**Table 1:** The potential therapeutic effects of Res in GC therapy

Cell line/Animal mod-el	Dose	Duration	Outcomes	Refs.
Human gastric cancer cell lines SGC7901 and BGC823	0, 5, 10, 25, 50, 100, 200 and 400 µM	24, 48 and 72 h	Inhibition of MALAT1-induced EMT	[63]
Human gastric cancer SGC-7901 cell line	0, 100, 200, 300 and 400 µmol/L	48 h	Suppressing Hh signaling pathway is associated with EMT inhibition	[93]
SGC7901 cells	35.69 µM	72 h	Administration of Res stimulates apoptotic cell death and cell cycle arrest in GC cells	[99]
Human gastric cancer SNU-1 cells	0, 10, 50 and 100 µM	24 h	Induction of apoptosis and reduced viability of cancer cells	[100]
Human gastric adenocarcinoma SGC7901 cells	0, 25, 50, 100 and 200 µmol/L	48 h	Stimulation of apoptotic cell death and DNA damage through enhancing the ROS production	[101]
Human GC cell lines AGS Nude mice xenograft model	0, 5, 10, 25, 50, 100 and 200 µM 40 mg/kg	24 h 4 weeks	Induction of cell cycle arrest and senescence	[110]
Balb/c-nu/nu mice BGC823 cells	0.1, 1, 5, 10, 20, 50 and 100 µg/ml 10 mg/kg	24 h 3 days	A significant reduction in tumor burden and an increase in apoptosis	[111]
Human gastric cancer SGC-7901 cell line	0, 100, 200, 300 and 400 µmol/L	48 h	Suppressing Hh signaling pathway is associated with EMT inhibition	[93]
SGC7901 cells	35.69 µM	72 h	Administration of Res stimulates apoptotic cell death and cell cycle arrest in GC cells	[99]
SGC-7901 cells	50, 200 and 400 µM	24 h	Induction of apoptosis by down-regulation of NF-κB	[112]
Human gastric cancer cell lines	0, 10, 20, 30, 40, 50 and 100 µM	48 h	Inhibition of IL-6-induced Raf-MAPK	[113]
Human gastric cancer cell lines that were either sensitive or resistant to cytostatic drugs	30 and 50 µM	72 h	Inhibition of MDR by down-regulation of ABCB1, P-gp, ANXA1 and TXN	[114]
Human gastric carcinoma SGC-7901 cells Nude mice	25 and 50 µM 50 mg/kg	24 h 21 days	Stimulation of apoptotic cell death in GC cells through mitochondrial pathway	[115]
Human gastric cancer cells SGC7901 and MGC803 Nude mice inoculated subcutaneously with SGC7901/DOX cells	50 mg/L 50 mg/kg	48 h 4 weeks	Res activates PTEN to down-regulate Akt resulting in EMT-mediated drug resistance	[168]
Human gastric adenocarcinoma cell line MGC803	0, 50, 100 and 200 µM	24 h	Inhibition of PI3K/Akt signaling pathway through PTEN down-regulation significantly induces cell cycle arrest	[167]

#### Resveratrol effect on multidrug resistance in chemotherapy

One of the most important difficulties faced in cancer therapy is multidrug resistance (MDR) [158–160]. MDR remarkably reduces the efficacy of chemotherapy [161, 162]. ATP binding cassette subfamily B member 1 (ABCB1) is one of the genes involved in MDR that by encoding P-glycoprotein (P-gp) inhibits the entering of anti-tumor drugs into cells [163–166]. Annexin A1 (ANXA1) and thioredoxin (TXN) are other possible mechanisms involved in MDR and consequently, cancer progression [167–169]. Administration of Res effectively diminishes the expression of ABCB1, P-gp, ANXA1 and TXN to suppress MDR [170]. [171]. Doxorubicin (DOX) is one of the potential chemotherapeutic agents with high capability in reducing the viability of cancer cells [172]. However, resistance to DOX treatment is a common phenomenon. It has been shown that PTEN is involved in EMT-mediated drug resistance [82]. Res is able to inhibit DOX resistance by stimulation of PTEN. The activated PTEN significantly diminishes Akt signaling pathway resulting in suppressing EMT-mediated drug resistance [173].

#### Resveratrol‐loaded drug delivery systems

There are a number of properties associated with mesoporous silica NPs (SLNs) making them suitable for delivery of genes and drugs [174]. These features include low particle size, sustained-release manner and large surface area [175]. This has led to the development of anti-miR-21- and Res-loaded SLNs for GC therapy. MiR-21 is an oncogenesis miR that significantly enhances the malignancy and invasion of cancer cells [176]. It seems that loading a combination of Res and anti-miR-21 on SLNs remarkably induces apoptotic cell death in GC cells. Besides, the synergistic impact of anti-miR-21 and Res reduces tumor burden [177], showing their efficacy in GC therapy.

## Conclusion and remarks

**Fig. 1:**
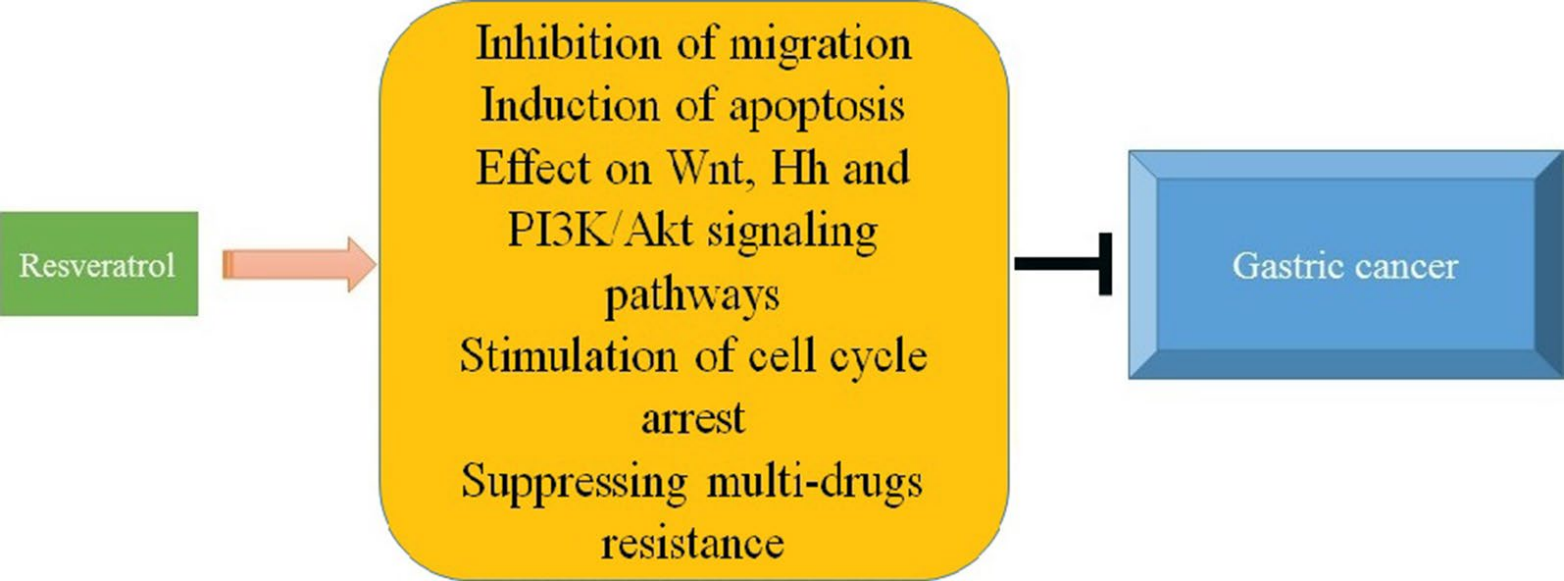
Application of Res in GC therapy.* Hh* hedgehog, * GC* gastric cancer, *PI3K* phosphatidylinositide 3-kinase

Taking everything into account, it seems that GC is still one of the most challenging disorders and there have been much effort to treat it. It is worth mentioning that cancer cells are able to obtain resistance to anti-tumor drugs. This urges scientists to develop novel anti-tumor drugs. However, it appears that synthetic anti-tumor drugs have high cost with a number of adverse effects against normal cells. Hence, plant-derived chemicals are of interest in cancer therapy. Res is a non-flavonoid polyphenol with several effects including apoptosis, cell proliferation inhibition, anti-inflammatory aspects. Several research has shown the therapeutic effects of Res for the amelioration of CRC and GC. Res has the potential preventive importance in gastric cancer. Res with several potential effects, is comparatively safe as well as able to target several cell signaling pathways. On the other hand, the bioavailability of Res seems to be very low in humans and due to the metabolic characteristics of res, even a high dose may not reach a sufficient concentration of treatment. Res may be of benefit for treatment of gasteric cancer. However, different techniques have been originated to increase the bioavailability of Res, more research are needed to differ the efficacy of Res in gastric cancer. Res is a non-flavonoid polyphenol with great anti-tumor activity. In the present review, we discussed the latest studies about the efficacy of Res in GC therapy. First off, it is noteworthy that nanocarriers are promising candidates in cancer therapy and due to the low bioavailability of Res, loading this compound on nanocarriers improves its anti-tumor activity. The metastasis of GC cells is a challenge and using Res is associated with reduced migration of GC cells through EMT inhibition. Chemotherapeutic agents are able to diminish the viability and proliferation of cancer cells through induction of apoptotic cell death. Res applies same strategy in GC therapy. Targeting Wnt signaling pathway is another capability of Res. By inhibition of Wnt, Res remarkably reduces the invasion of GC cells. Besides, Res is capable of targeting PI3K/Akt and Hh signaling pathways in GC therapy. More importantly, administration of Res enhances the potential of chemotherapy by sensitizing tumor cells (Fig. 1). These significant anti-tumor effects of Res make it an appropriate choice for treatment of GC. Importantly, urging scientists to investigate the potential anti-tumor activity of Res against GC in clinical trials in of interest. A look into clinicaltrials.gov demonstrates that Res is able to prevent cancer progression and recurrence. Unfortunately, there is no study regarding the anti-tumor activity of Res against GC in clinical trial. This should be considered at the next studies.

## Data Availability

The datasets used and/or analyzed during the current study are available from the corresponding author on reasonable request.

## Abbreviations

Res: Resveratrol
STS: Stilbene synthase
UV: Ultra-violet
uPAR: Urokinase-type plasminogen activator receptor
EGFR: Epidermal growth factor receptor
OSCC: Oral squamous cell carcinoma
miR: MicroRNA
EMT: Epithelial-to-mesenchymal transition
WHO: World Health Organization
CT: Computed tomography
MRI: Magnetic resonance imaging
GC: Gastric cancer
lncRNA: Long non-coding RNA
MAPK: Mitogen-activated protein kinase
Nrf2: Nuclear factor erythroid 2-related factor 2
5-FU: 5-fluorouracil
NPs: Nanoparticles
SLNs: Mesoporous silica NPs
MALAT1: Metastasis-associated lung adenocarcinoma transcript 1
NF-KB: Nuclear factor-KB
IL-6: Interleukin-6
MDR: Multidrug resistance
ABCB1: ATP binding cassette subfamily B member 1
P-gp: P-glycoprotein
ANXA1: Annexin A1
TXN: Thioredoxin
DOX: Doxorubicin
GSK-3β: Glycogen synthase kinase-3β
Hh: Hedgehog
Sirt1: Sirtuin 1
PKC: Protein kinase C
NO: Nitric oxide
NOS: Nitric oxide synthase
